# Arterial Bullet Embolization Secondary to Direct Cardiac Injury in Penetrating Chest Trauma: A Rare Challenging Case Report

**DOI:** 10.7759/cureus.19033

**Published:** 2021-10-25

**Authors:** Kiarash Mashayekhi, Abdul Waheed, Frank R Kennedy

**Affiliations:** 1 Trauma, San Joaquin General Hospital, French Camp, USA; 2 Surgery, San Jaoquin General Hospital, French Camp, USA; 3 Surgery, San Joaquin General Hospital, French Camp, USA

**Keywords:** high velocity, trauma, pulmonary trauma, chest wall trauma, penetrating cardiac injury, bullet embolism

## Abstract

Severe thoracic injury secondary to penetrating trauma requires prompt resources and rapid decision-making by trauma centers and teams. Implementing trauma systems has significantly impacted medical and critical care quality and outcomes, including managing rare trauma injuries. We describe a report of a rare case of a 21-year-old man with a gunshot wound to the chest with injuries to the right pulmonary hilum requiring pneumonectomy and to the left atrium with bullet embolism to the right common iliac artery. In addition, the systematic approach where each phase of the individual's treatment -- prehospital, emergency room, running room, and intensive care -- was positively affected by the implementation, development, and progressive maturation of a trauma system is also explained.

## Introduction

Patients presenting with severe thoracic injury secondary to penetrating trauma require immediate resources and rapid decision-making by trauma centers and teams [1,2]. Implementing trauma systems has significantly impacted the quality and outcomes of medical and critical care, including managing rare trauma injuries [3,4]. Bullet embolus remains a rare entity, and a high index of suspicion must be present to identify a bullet embolus and should be considered when discrepancies between the number of entry and exit wounds exist and in signs of peripheral ischemia [5,6]. Further, this "single case continuum of care review" may be a valuable tool that outside reviewers can utilize to evaluate the care of a trauma system and trauma center. Here we report a rare case of a 21-year-old man with a gunshot wound (GSW) to the chest with injuries to the right pulmonary hilum requiring pneumonectomy and the left atrium with bullet embolus to the right common iliac artery. Further, the ways whereby each phase of the patient's care - prehospital, emergency room (ER), operating room (OR), and intensive care - was positively impacted by the implementation, evolution, and progressive maturation of a trauma system are documented and discussed.

## Case presentation

**Figure 1:**
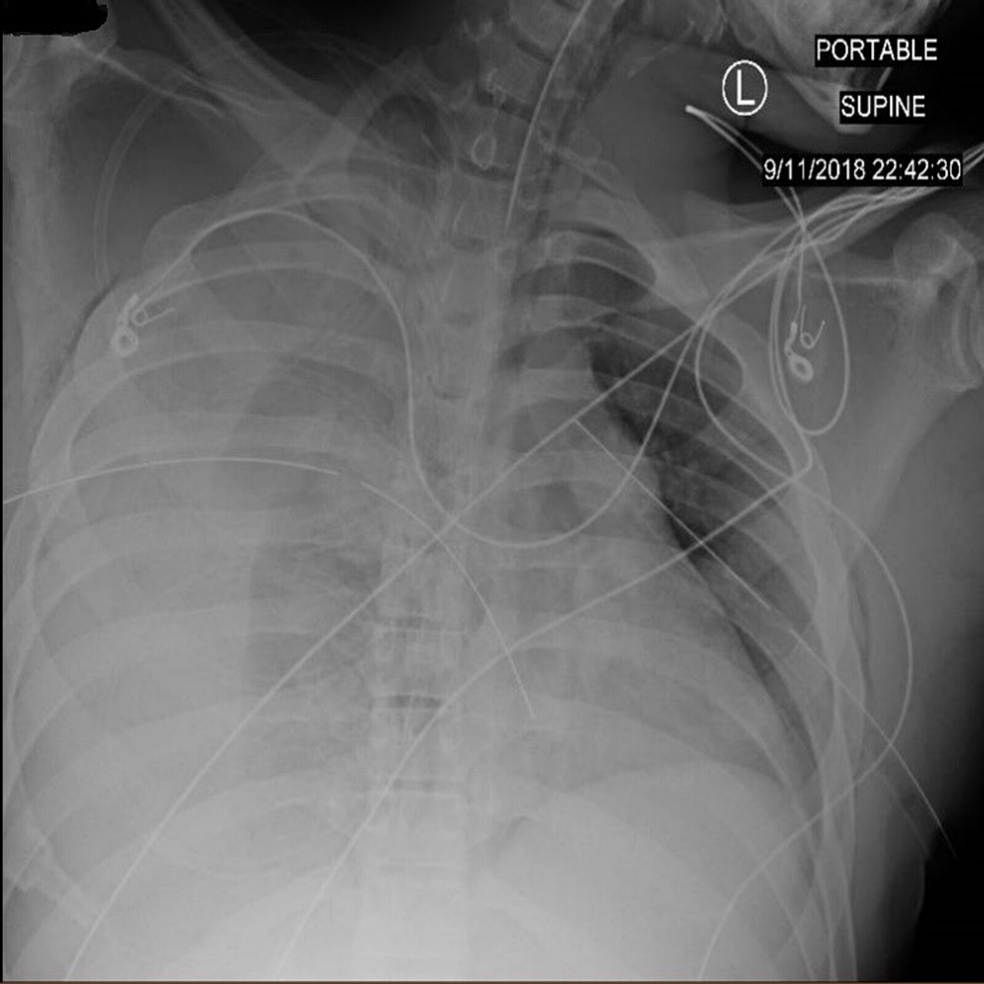
Trauma bay portable X-ray after intubation and placement of bilateral chest tubes

A 21-year-old man with a 9-millimeter (mm) caliber GSW to the right axilla and a Glasgow Coma Scale of 8, decreased breath sounds, and feeble pulses was brought to the ER. He was quickly intubated, and bilateral chest tubes were placed with an instant 700 mL of blood evacuation (Figure 1).

**Figure 2:**
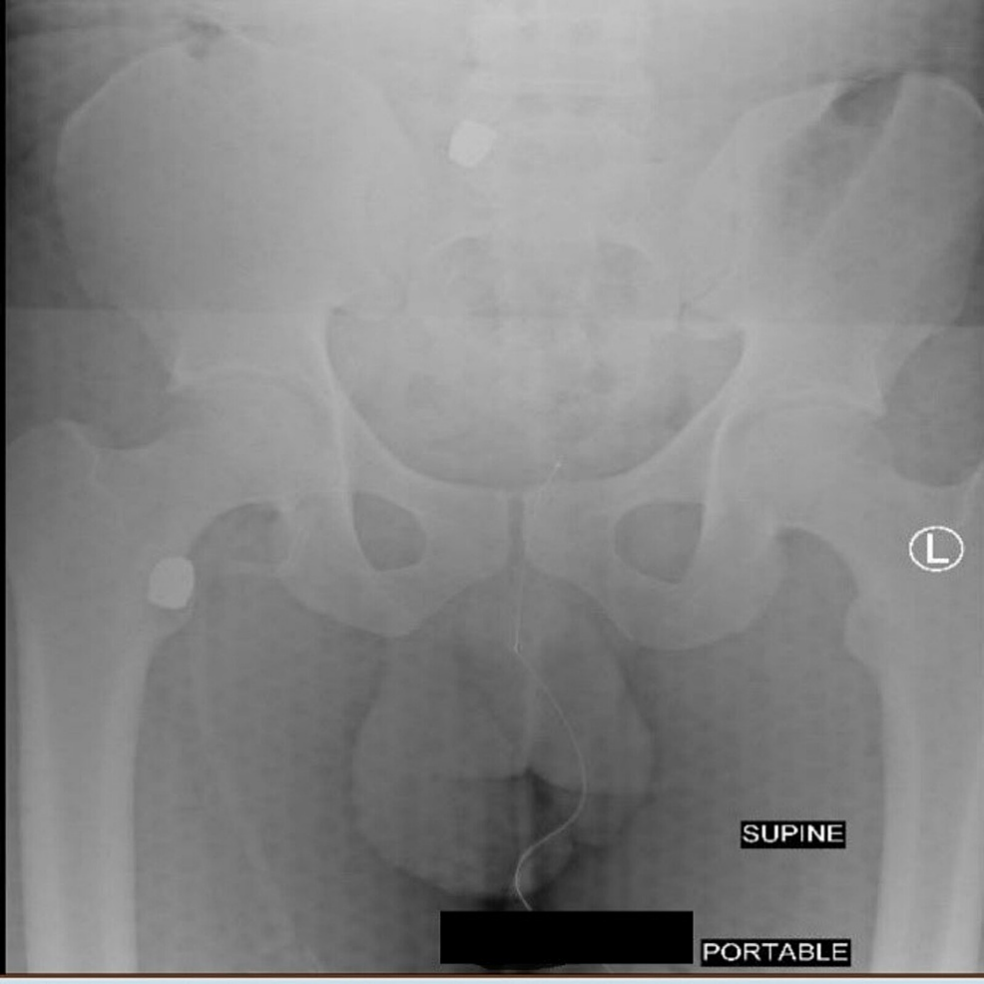
Postoperative KUB demonstrating two bullets in the RLQ and right proximal femur KUB, kidney, ureter, and bladder; RLQ, right lower quadrant

A right femoral cordis line was established, and massive transfusion protocol was initiated. The Focused Assessment with Sonography in Trauma (FAST) examination revealed fluid in Morrison's pouch. A chest X-ray (CXR) showed a large right hemothorax, and he was instantly transferred to the OR. A right thoracotomy was done, which showed significant injury to the right pulmonary hilum and tense hemopericardium. The pericardium was opened, blood evacuated, and a digital examination of the heart was performed, which revealed left atrial injury, and the bleeding was controlled with a finger. The incision was then extended to the left thorax to improve exposure. The right hilum was clamped, and the left atrial injury was ultimately controlled with a Foley catheter occlusion. Attention was then turned to the pulmonary hilum, where local control of bleeding attempts failed, and pneumonectomy was performed. Moreover, the physical examination in the OR revealed inability to feel the pulses in the right lower extremity. An exploratory laparotomy was then done, which was negative, and he was transferred to the surgical intensive care unit (SICU). In the SICU, he had non-palpable pulses (dorsalis pedis, popliteal, and femoral), and his creatinine kinase was found elevated to a level of 150,000 u/L. A postoperative kidney, ureter, and bladder imaging revealed two bullets in the right lower quadrant and right proximal femur, and he was taken back to the OR (Figure 2). 

**Figure 3:**
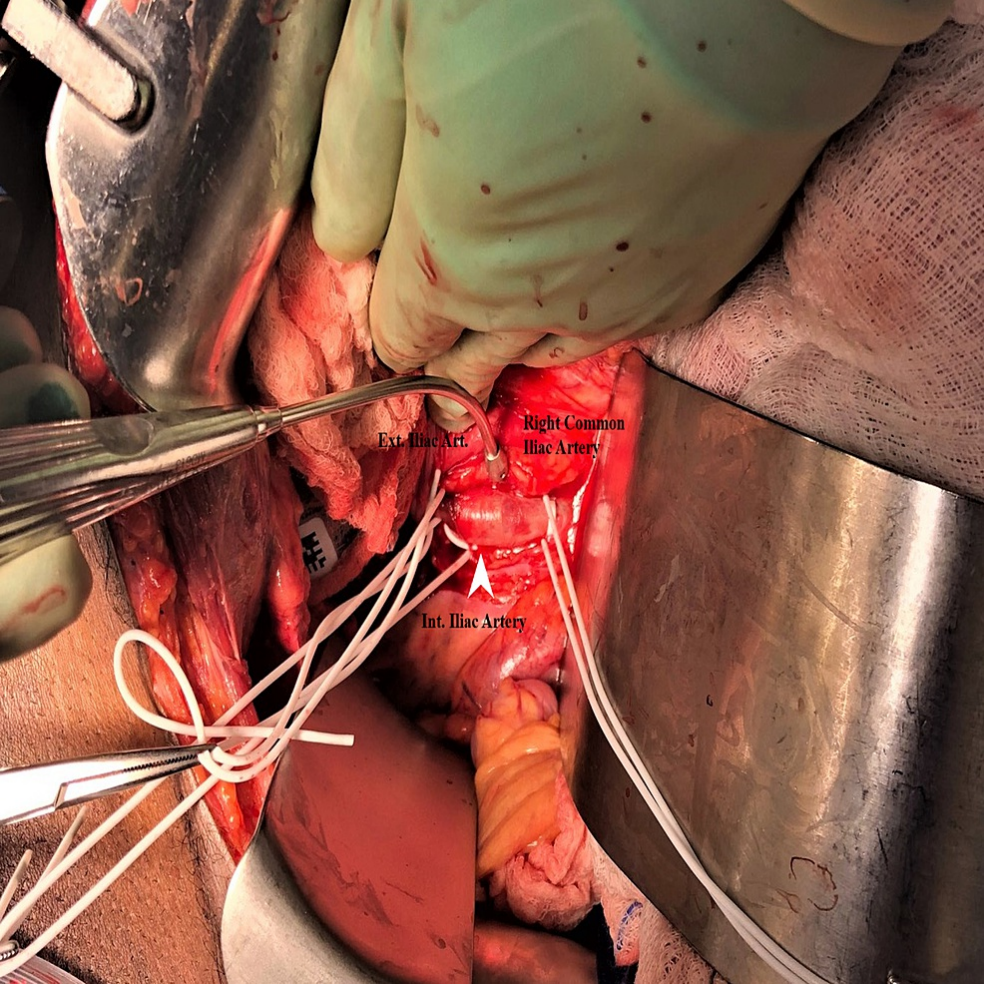
Exposure of the right common Iliac artery. Proximal and distal control was obtained. Bullet (white arrow) lodged at the bifurcation with occlusion of the right internal and external iliac arteries

A midline laparotomy incision was reopened, and the right common iliac artery was explored, revealing the bullet embolus in the distal right common iliac artery (Figure 3). 

**Figure 4:**
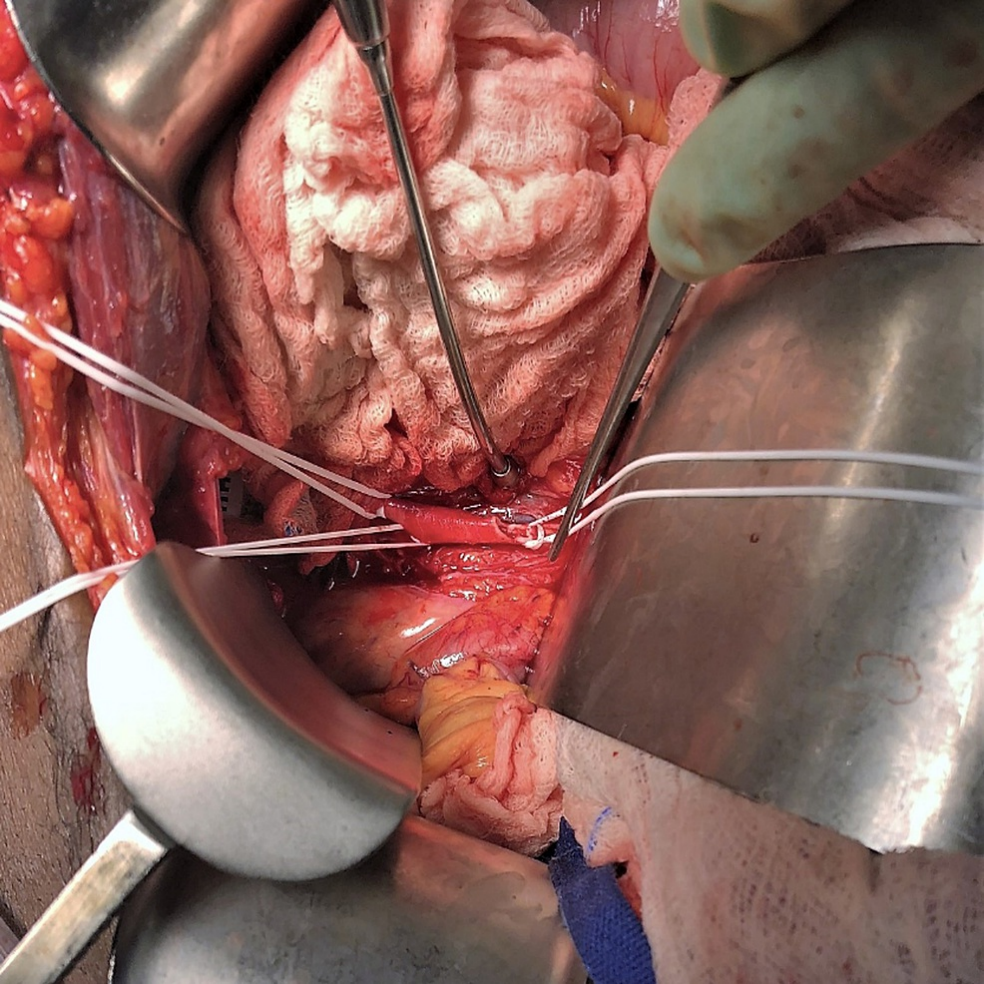
Transverse arteriotomy at the level of right common Iliac artery, and retrieval of the bullet

**Figure 5:**
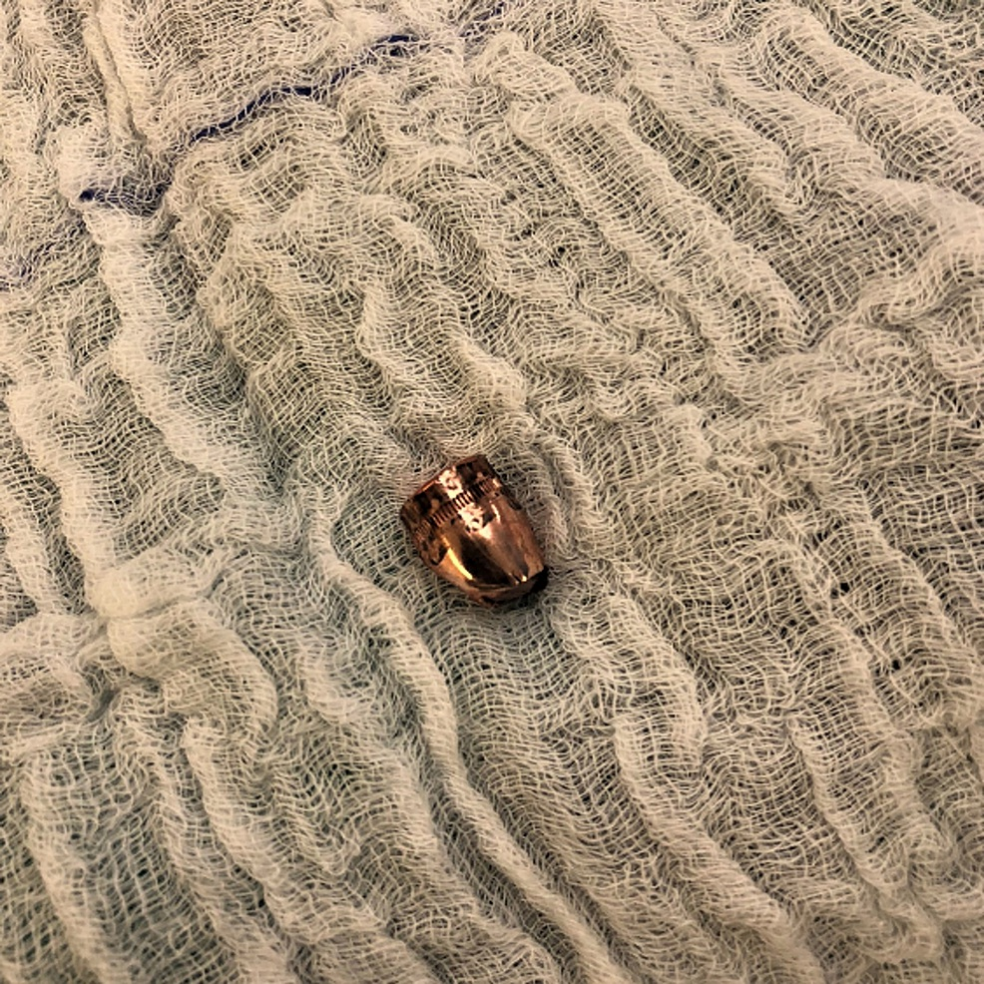
Extracted bullet from the right common iliac artery

The distal and proximal control was obtained, and the bullet was removed via a transverse arteriotomy (Figures 4, 5). 

Thrombectomy was performed, and the arteriotomy was closed. The patient then had a strong femoral pulse. Simultaneously, above the knee, guillotine amputation was performed. The abdomen was left open, and the patient was transferred to the SICU. In the SICU, the patient developed acute kidney injury, and underwent very early postoperative daily hemodialysis. Furthermore, he experienced myocardial infarction on postoperative day (POD) 1; however, he was effectively resuscitated. After some initial recovery, it was determined to close his abdomen on POD 2 in the SICU, which was uneventful, and on POD 5, he was off all pressor agents. Unluckily, shortly after the initial improvement, his condition rapidly deteriorated; he became hypoxemic, refractory to all rescue maneuvers, had another heart attack, and passed away afterward. 

## Discussion

First described by Thomas et al. in 1834, the intravascular bullet embolism following a direct cardiac injury after penetrating GSW to the chest is a rare phenomenon in both civilian and military trauma patients [7]. The true incidence of bullet embolism is hard to describe due to the nature of the injury. However, Rich et al. in 1978 reported an incidence of 0.3% annually in the USA, and Aidinian et al. reported an incidence of 1.1% in military soldiers combating in Iraq and Afghanistan [8]. Moreover, in 1998, Kennedy et al. published a scarce case of arterial bullet embolism to the innominate artery after GSW to the right heart and injury to the ventricular septum with subsequent downstream migration to the abdominal aortic bifurcation [9]. Likewise, the clinical presentation of the bullet embolism can often be concealed and deceitful. The disparity between the number of entry and exit wounds, physical examination findings, and radiological findings inconsistent with the bullet's trajectory must raise the possible suspicion of arterial bullet embolism [10]. The final consequences of the intravascular embolism also depend on the type of vessel involved. Most venous emboli are asymptomatic in almost 70-90% of the cases, while the arterial bullet emboli pose considerably more significant injury [10]. Besides the risk of ischemia and infarction, the arterial bullet embolism may result in dislodgement, erosion, further advancement in the vascular tree, distal and proximal thrombus formation, delayed vascular compromise, septicemia, and death [10]. With the high clinical suspicion, a proper physical examination, and imaging techniques like CXR, computed tomography (CT) imaging, and transthoracic and transesophageal echocardiographic imaging, the precise diagnosis can be made in most cases [7,8,10]. Moreover, the guidelines on the accurate management of arterial bullet embolization remain controversial. Most authors recommend a conservative approach to small asymptomatic intravascular arterial bullet embolization, but this is debatable [7,10]. However, removing the bullet with adjunctive surgical procedures is inevitable in symptomatic arterial bullet embolization [7]. All the symptomatic visceral and peripheral arterial bullet emboli should be removed as soon as possible to prevent further extension of the injury [8]. In cases where significant damage is already done, there is a low likelihood of any other damage, and in the unstable patient, the embolus can be left in place. In case of distal emboli to the extremities, the possibility of reperfusion injury and protective fasciotomies must be considered [10]. 

## Conclusions

Arterial bullet embolus is an infrequent phenomenon noticed in the trauma world. A high index of suspicion must be present to identify arterial embolus and should be considered when discrepancies occur between the entry and exit wounds and in signs of peripheral ischemia. 
